# Gangrene

**Published:** 1890-04-26

**Authors:** 


### GANGRENE.

Dr. O'Neil reports a case of gangrene of the leg, preceded by phlegmasia dolens, in a lady eighty years of age. She was attacked with great pain in the calf, and swelling of the whole extremity. The large vein in the thigh was tense and painful. Under treatment the swelling disappeared. Soon afterwards she was attacked with violent pain in the leg and foot, which looked as if a "blue silk stocking" had been drawn on, with several holes in it. On the instep especially, dark red œdematous skin protruded. Under stimulants there was some improvement, but the blue colour extended up the thigh, and the case terminated fatally, with exhaustion, diarrhœa, and delirium. Dr. O'Neil believes the first attack was caused by an obstruction in the femoral vein, and the second by an obstruction in the popliteal or lower part of the femoral artery. The obstruction to the venous blood in its course to the heart and lungs caused the extremity to swell in all directions, nevertheless the limb recovered. The principal vein in a limb may be obstructed, and its functions interfered with, without danger to life, until a collateral circulation becomes established. But obstruction of a large artery is a more serious matter, for the supply of blood being cut off, collapse, starvation, and death of the tissues follows. The last fatal symptoms may have been the result of septic fluid from the diseased part escaping into the general circulation.

